# WeedNet-R: a sugar beet field weed detection algorithm based on enhanced RetinaNet and context semantic fusion

**DOI:** 10.3389/fpls.2023.1226329

**Published:** 2023-07-24

**Authors:** Zhiqiang Guo, Hui Hwang Goh, Xiuhua Li, Muqing Zhang, Yong Li

**Affiliations:** ^1^School of Electrical Engineering, Guangxi University, Nanning, China; ^2^Guangxi Key Laboratory of Sugarcane Biology, Guangxi University, Nanning, China

**Keywords:** precision farming, deep learning, object detection, weed recognition, sugar beets

## Abstract

Accurate and dependable weed detection technology is a prerequisite for weed control robots to do autonomous weeding. Due to the complexity of the farmland environment and the resemblance between crops and weeds, detecting weeds in the field under natural settings is a difficult task. Existing deep learning-based weed detection approaches often suffer from issues such as monotonous detection scene, lack of picture samples and location information for detected items, low detection accuracy, etc. as compared to conventional weed detection methods. To address these issues, WeedNet-R, a vision-based network for weed identification and localization in sugar beet fields, is proposed. WeedNet-R adds numerous context modules to RetinaNet’s neck in order to combine context information from many feature maps and so expand the effective receptive fields of the entire network. During model training, meantime, a learning rate adjustment method combining an untuned exponential warmup schedule and cosine annealing technique is implemented. As a result, the suggested method for weed detection is more accurate without requiring a considerable increase in model parameters. The WeedNet-R was trained and assessed using the OD-SugarBeets dataset, which is enhanced by manually adding the bounding box labels based on the publicly available agricultural dataset, i.e. SugarBeet2016. Compared to the original RetinaNet, the *mAP* of the proposed WeedNet-R increased in the weed detection job in sugar beet fields by 4.65% to 92.30%. WeedNet-R’s average precision for weed and sugar beet is 85.70% and 98.89%, respectively. WeedNet-R outperforms other sophisticated object detection algorithms in terms of detection accuracy while matching other single-stage detectors in terms of detection speed.

## 1 Introduction

Damage caused by weeds on fields is a significant factor influencing agricultural progress. Weeds in the field compete with crops for sunshine, water, and nutrients, resulting in a deterioration in crop quality and a fall in crop output, which causes substantial losses to the agricultural economy. With the rapid development of agricultural mechanization and information technologies, it is anticipated that automatic weeding robots will be widely applied in weed management, achieving the goals of reducing pesticide use, conserving resources, protecting the ecological environment, and increasing agricultural yields. Vision-based weeding robots for weed management rely heavily on the detection and identification of weeds (Li et al., 2022). Complex farming landscapes with dynamically changing, unstructured, and various conflicting noise characteristics make it challenging for weeding robots to detect and find weeds in the field. In addition, the diversity of weed morphology at various growth phases and the complexity of the soil background in which weeds grow aggravate the difficulties of weed detection. Consequently, weed detection and localization in the field remains a difficult undertaking (Wang, 2019).

In recent years, significant progress has been made in machine-vision-based weed detection approaches. However, the field of weed detection on farmland still faces persistent challenges, including the scarcity of available weed datasets, the presence of monotonous backgrounds, limited availability of diverse learning samples, the inability to achieve end-to-end solutions, and low detection accuracy. These challenges continue to pose obstacles for researchers and practitioners in the field. To address the aforementioned challenges and foster the advancement of deep academic-based target detection technology in the field of weed detection on farmland, we reconstructed an weed dataset of about 5000 images with annotation labels of bounding boxes based on the publicly available agricultural dataset SugarBeets2016 and named it OD-SugarBeets. In the meantime, we present WeedNet-R, an object identification network based on the one-stage framework network RetinaNet, for weed recognition and localisation in sugar beet fields. Inspiring by the work of Najibi et al. (2017) and Deng et al. (2019) on face detection, WeedNet-R incorporates numerous context modules in the neck of RetinaNet to combine feature maps with varying receptive field sizes from distinct layers. Utilizing context modules improves the WeedNet-R’s capacity to represent context information, hence enhancing its weed identification precision. Moreover, a learning rate adjustment method combining an untuned exponential warmup schedule and cosine annealing technique for the Adam optimizer is implemented during model training in order to increase the network’s ability to seek for its global optimal solution. In addition, we present a crop-first non-maximum suppression strategy to eliminate repeated prediction bounding boxes below a certain confidence level. The object that is anticipated by the network to be both weeds and crop is favored to be crop to minimize the possibility of crop being erroneously removed.

The following is a summary of this article’s primary contributions. (1) We propose the WeedNet-R weed detection model for sugar beet fields, which is based on RetinaNet. Multiple context modules are added to WeedNet-R’s neck in order to expand the network’s receptive field. As a result, the accuracy of weed recognition is enhanced without a major increase in model parameters. (2) An untuned exponential warmup schedule is set for the Adam optimizer during WeedNet-R training, thereby enhancing the network’s search potential for global optimal solutions. (3) Nearly 5,000 images from the SugarBeet2016 dataset were manually re-labeled with bounding boxes to address the limitation that the dataset cannot be utilized directly to object detection techniques. The SugarBeet2016 update dataset has been published to a public repository^1^ for the development and assessment of other weed algorithms.

The remaining sections are organized as follows. In Section 2, the relevant works on deep learning-based weed detection systems from recent years are briefly discussed. In Section 3, the picture dataset and proposed method for weed detection will be introduced. Section 4 describes the experimental conditions and associated assessment metrics for weed detection. Section 5 contains the entire experimental findings analysis and commentary. Finally, in Section 6 we end our task.

## 2 Related works

In recent years, the field of weed detection has witnessed a growing interest in deep learning and image recognition-based approaches. Within this context, two main machine vision-based strategies have emerged: individual or pixel-level classification and object detection or instance segmentation.This section provides a comprehensive review of individual or pixel-based classification methods and object detection or instance-based segmentation methods. Subsequently, we provide concise definitions of key concepts related to network enhancements, including context information, focus loss, and warmup schedule.

### 2.1 Individual or pixel level classification-based methods

Individual level classification-based approaches use the entire image as the model input and differentiate between weeds and crops based on the classification of the image. This approach has been widely employed in weed detection investigations in the past. Olsen et al. (2019) employed Inception-v3 and ResNet-50 as baseline models to test weed classification performance on the DeepWeeds public images dataset (https://github.com/AlexOlsen/DeepWeeds). The average classification accuracy of these models is 95.1% and 95.7%, respectively. Hu et al. (2020). developed graph convolution to characterise RGB images as multi-scale graphs in order to generate deep feature representations at a fine-grained level, and the average classification accuracy on DeepWeeds was 98.1%. Espejo-Garcia et al. (2020) integrated convolutional neural networks with standard machine learning classifiers in order to capitalise on the powerful feature extraction capabilities of convolutional neural networks and the high classification performance of machine learning classifiers. Consequently, the DenseNet-SVM model earned an F1 score of 99.29% on the picture dataset of various Greek farms.

Typically, the pixel level classification-based algorithms categorise each pixel in the detected image into one of three categories: crop, weed, and background, thereby separating weed and crop from the background. Recent investigations have been undertaken on the basis of this concept. (Lottes et al., 2016) suggested an encoding-decoding model based on fully convolutional networks (FCN) to distinguish crop and weed from the background by including spatial information from image sequences. Sa et al. (2017) proposed a pixel-wise segmentation network named ‘weedNet’ based on SegNet (Badrinarayanan et al., 2017) to classify weeds and crops in UVA’s images. And Bosilj et al. (2020) included transfer learning into SegNet for weed recognition in various types of crops to reduce the necessary retraining time and labeling effort for new crop types. Image segment improvement techniques have also attracted the interest of researchers. By combining NIR image information, Wang et al. (2020) increased the resilience of segmentation algorithms against diverse lighting situations. In their work, the best mean intersection over union (mIoU) for pixel-wise segmentation was 88.91%. In addition, Fawakherji et al. (2019). employed a deep network based on the UNet for pixel-wise semantic segmentation, background removal, and ROIs extraction. A CNNs-based classifier was then applied to classify the retrieved ROIs as crop or weed. However, neither individual-level classification nor pixel-level classification can simultaneously classify and locate weeds end-to-end. And they require image additional pre-processing and post-processing techniques to detect the distribution of weeds and crops in the images.

### 2.2 Object detection or instance segmentation-based methods

Unlike individual or pixel-level classification-based methods, object detection-based methods for weed detection discover all objects of interest using prediction bounding boxes including category information. In recent years, object detection approaches based on deep learning have garnered increasing interest for weed detection and location on agriculture. Jiang et al. (2019) suggested a two-stage network with Inception-ResNet v2 as the backbone based on Faster R-CNN and transfer learning to detect in-row weeds in cotton fields. However, the quantity of weeds in the image datasets used was very limited, making detection easier. Gao et al. (2020) suggested a data augmentation approach for training samples and combined synthetic and original field images to train the YOLOv3-based model, which produced a *mAP* of 0.829%. However, the dataset utilized was quite limited and contained a monotonous soil background, and the strategy of increasing the original dataset via picture synthesis alone could result in model overfitting. Jin et al. (2021) suggested a system based on deep learning to detect weeds in vegetable fields. A trained CenterNet model was initially used to locate vegetable plants with bounding boxes. Then, image segmentation was utilized to identify weeds outside the vegetable-bounding boxes. It is evident that this detection method is not end-to-end, as the complicated image post-processing will require a significant amount of CPU resources. In addition, the color-index-based picture segmentation method is highly sensitive to illumination and plant colour, therefore the algorithm’s capacity for generalization may be limited. Zhuang et al. (2022). assessed the effectiveness of five distinct object identification models for the detection of broadleaf weeds in wheat seedlings. Since none of these models have a recall rate more than 0.58, the researchers concluded that these models are insufficient to detect weeds in wheat without improvement. In a new study, researchers are investigating a method based on instance segmentation for detecting the contours and locations of weeds in images of farmland. For instance, Champ et al. (2020) trained and evaluated a Mask R-CNN model for field weed detection using a data set containing 2489 image samples, achieving a pretty good detection accuracy. In actual weed control, weed eradication efficiency reached up to 60 percent. However, instance segmentation-based methods for weed detection demand more computing resources than object detection-based methods.

Recently, Transformer has shown great success in Natural Language Processing (NLP). It has also been applied to computer vision tasks, yielding excellent results (Dosovitskiy et al. 2021). Transformer-based object detectors, such as Swin-Transformer (Liu et al., 2021), DETR (Zhu et al, 2020), and DINO (Zhang et al., 2022a), have emerged and been applied in weed detection tasks(Zhang et al., 2022b).

### 2.3 Context information

**Figure 1:**
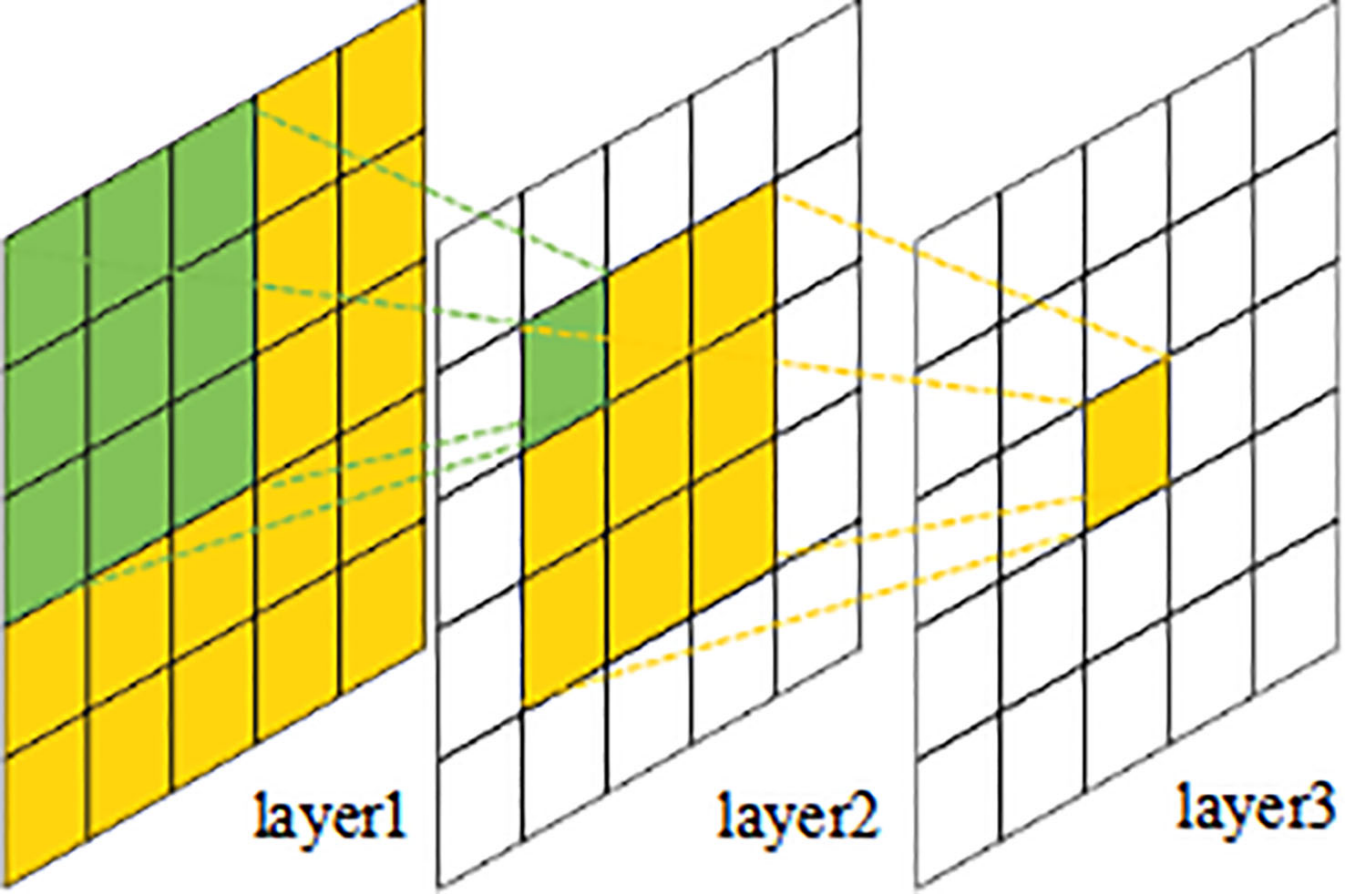
Receptive field sizes of different layers.

In a convolutional neural network, the receptive field (RF) represents the capacity of the convolutional unit to sense the size of the input region. Typically, the receptive field size is calculated beginning with the first layer of the input feature map, and different convolutional layers have varying receptive field sizes. As demonstrated in **Figure 1**, the theoretical receptive field (TRF) of a convolutional neural network increases as the number of convolutional layers increases in depth. The greater the value of RF, the larger the region of the raw input that the output feature map sees, which may imply more global and higher-level semantic characteristics. However, for deep learning models, the effective receptive field (ERF) has a greater impact than the fixed TRF of the networks. In order to expand the ERF of the model, context information is utilized and enhanced by fusing the model’s characteristics with RFs of varying sizes from different layers. Najibi et al. (2017) accomplished more efficient contextual modelling by adding additional convolutional filtering layers to each prediction module of the SSH face detection network, hence obtaining a larger ERF. PyramidBox (Tang et al., 2018) has developed a context-aware prediction module that retains rich context information from multiple feature layers. Deng et al. (2019) introduced independent contextual modules to the five feature layers of the FPN of the single-stage face detector to raise the ERF of the network, hence enhancing its rigid context semantic modelling capabilities.

### 2.4 Focal loss

**Figure 2:**
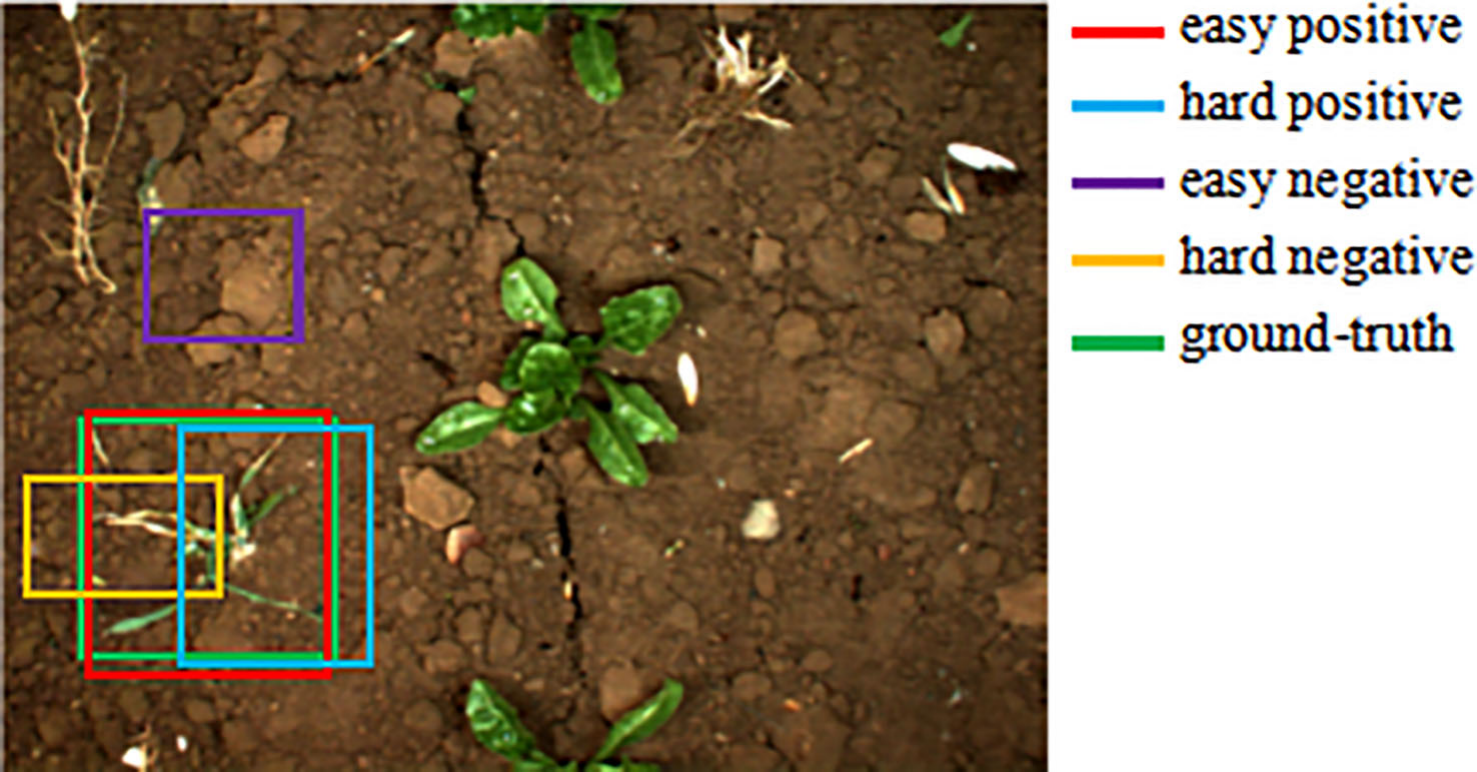
The distribution of different examples.

The majority of early classical object identification algorithms employed the cross entropy function as the classification loss of the object detection network. However, the weight of the conventional cross entropy loss function is the same for all instances (easy positive, hard positive, easy negative and hard negatives, as shown in **Figure 2**). In the case of example imbalance, a large number of simple negative cases will predominate, whereas a small number of hard positive and hard negative examples will not play a role, hence complicating model optimization during training. To address the issue of imbalanced examples during model training, Lin et al. (2020) presented the focal loss function, which focuses the model’s attention on the acquisition of challenging cases. As confidence in the proper class improves, the focal loss function introduces a dynamic scaling factor based on the cross-entropy function that decays to zero. As a result, this scaling factor can automatically down-weight the contribution of easy cases during training and fast centre the model’s attention on challenging examples. RetinaNet, a one-stage object detector able to match the speed of earlier one-stage detectors while surpassing the accuracy of all contemporary two-stage detectors, was used to test the effectiveness of focus loss.

### 2.5 Warmup schedule

Adam optimizer, an adjustable learning rate gradient descent method, has become increasingly popular in recent years for training models in deep learning due to its rapid convergence and great efficiency. Nonetheless, according to a recent study (Liu et al., 2020) the problematically huge variance of the adaptive learning rate in the early stage of model training is the primary reason of poor model convergence. Ma and Yarats (2021) demonstrate that even if the model is started to a local minimum, the Adam optimizer’s early parameters update may exhibit significant non-regularity. The most popular method for enhancing Adam’s stability is to include a warming schedule during model training to reduce significant or divergent variance (Liu et al., 2020; Ma and Yarats 2021). Typically, the warmup schedule is established during the first few epochs or partial steps of model training. During the warmup period, the learning rate is reduced to a low amount. The training with a low learning rate increases the likelihood that the randomly initialized model’s weights will stabilize.

## 3 Materials and methods

### 3.1 Weed dataset

**Figure 3:**
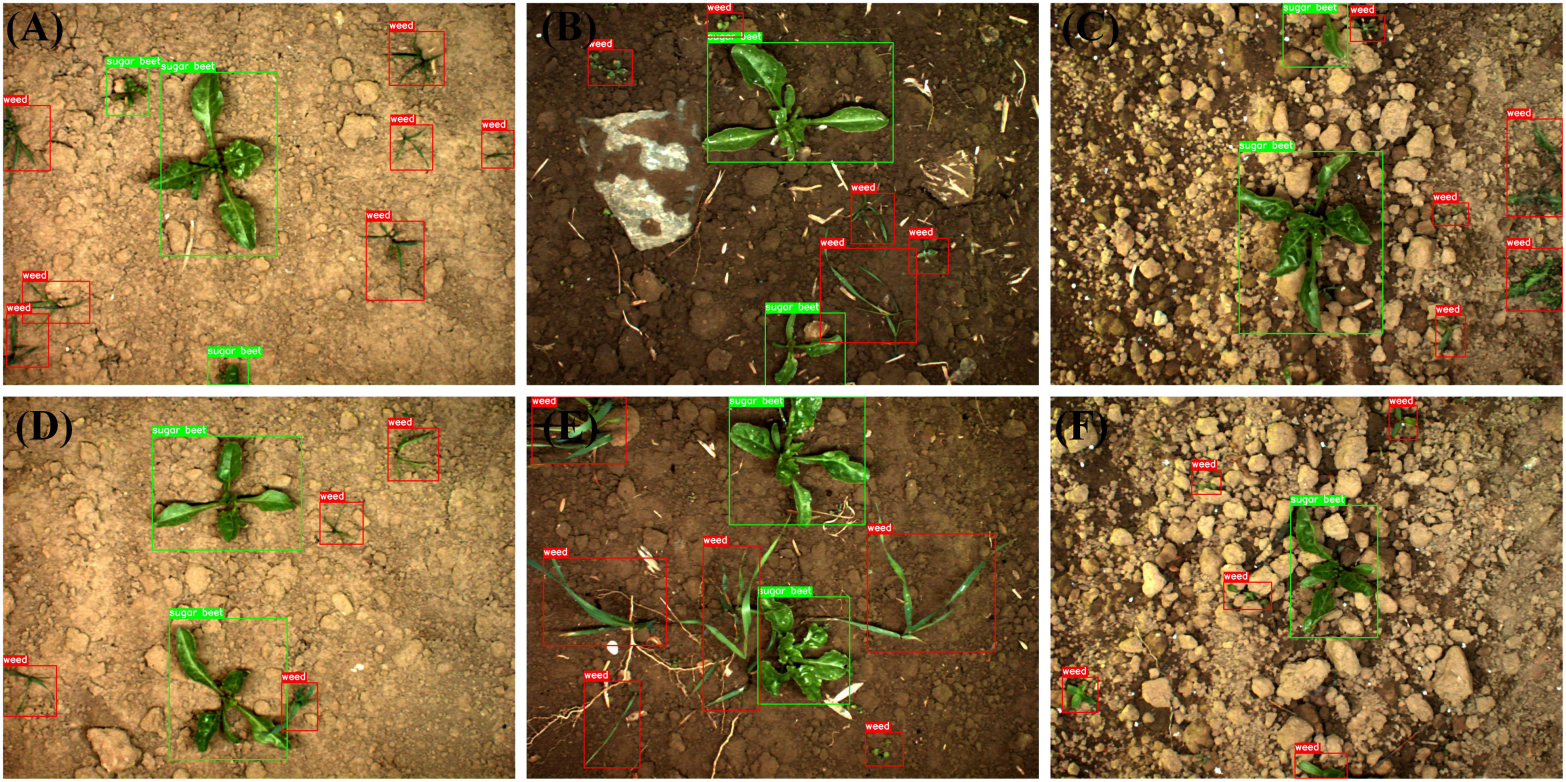
Sample images and annotations: **(A-C)** from training set, **(D-F)** from test set.

SugarBeets2016 (University of Bonn, Germany), a huge agricultural robotics dataset for weed classification, localization, and mapping, serves as the basis for our investigations (Chebrolu et al., 2017). The collection contains three months of data acquired by the BoniRob robotic platform from a sugar beet field near Bonn, Germany. The data is collected two to three times per week, on average, and covers the pertinent growth stages for robotic intervention and weed control. The RGB images of SugarBeets2016 were captured by the JAI AD-130GE multi-spectral camera mounted on the bottom of the BoniRob robot from a top-down perspective and saved in the PNG format with lossless compression and a uniform size of 1296 936 pixels. Since the time of weed management in sugar beet fields is typically during the rapid growth period of the sugar beet leaves, rather than during the crop seedling stage when the weed morphology is most comparable to that of the crop, the sugar beet leaves have a similar morphology to the weeds. As a result, the images from the period of rapid leaf growth were chosen as our experimental material, and these images were obtained 20 days after sugar beet growth began above ground. Using the labelme tool (https://github.com/wkentaro/labelme), all the items in the 4,817 images of farmland were categorized as either sugar beet or weed, as depicted in **Figure 3**. A human expert manually identifies all the objects inside the detected image and encloses the region containing these things with closed rectangular boxes; the category and location information of these objects are then recorded to a local XML file. There were 9,419 sugar beet items and 9,349 weed objects in the labelled dataset.

### 3.2 RetinaNet based weed and sugar beet detection model

#### 3.2.1 Context module

**Figure 4:**
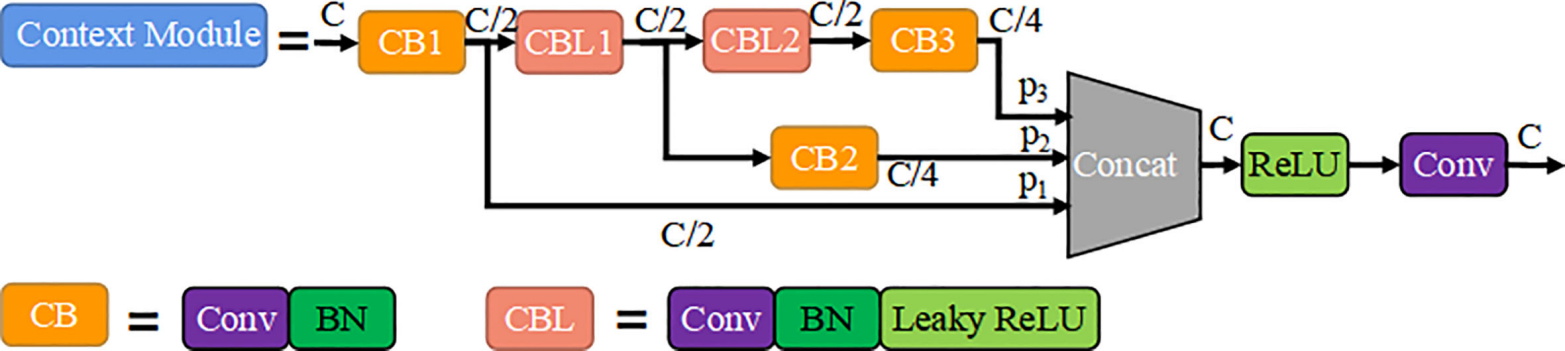
Context module.


As shown in **Figure 4**, the **c**ontext module contains four convolutional layers, where the coefficient of ‘*C*’ represents the number of input or output channels of convolutional layer. *Conv-k* denotes a convolution layer with s stride size of 1 and a kernel size of *k*×*k* (default is 3×3), *BN* is bath normalization, *ReLU* denotes activation function. The *CB* block indicates the addition of bath normalization after the convolutional output, and *CBL* block indicates the addition of the *Leak ReLU* activation function to the *CB* module. The branch *y_2_* consisting of *CBL1* and *CB2* has a total stack of 2 convolutional layers, so the output of this layer has a receptive field size relative to the input equal to the receptive field size of a 5×5 convolution. Similarly, the branch *y_3_* consisting of *CBL1*, *CBL2* and *CB3* has a stack of *3* convolution layers, so the size of receptive field of this layer is equal to the 7×7 convolution layers. The *Y_j_* =[*y_1_*, *y_2_*, *y_3_*] are calculated as shown in Equation (1), where $f_{c}\left(\cdot \right)$ is a convolutional operation with a kernel size of 3×3 and a step size of 1, $f_{cb}\left(\cdot \right)$ is the use of batch normalization after $f_{c}\left(\cdot \right)$ . $f_{cbl}\left(\cdot \right)$ is the addition of Leaky ReLU activation function on top of $f_{cb}\left(\cdot \right)$ . The convolutional layer outputs *Y_j_* obtained from the input feature maps after convolutional operations in different layers are finally fused with semantic information of different scale by a concatenation method. Suppose *P_i_* =[*P_3_*,*P_4_*,*P_5_*] are the feature maps from FPN, and ${\dot{P}}_{i}=[{\dot{P}}_{3},{\dot{P}}_{4},{\dot{P}}_{5}]$ are the feature outputs after a context module, *P_i_*, $\;{\dot{P}}_{i}\;\epsilon \;R^{H_{i}\times W_{i}}$ , $\;H_{i}\times W_{i}$ is the size of feature map *P_i_*. As a result, *V*_i_ is calculated be the Equation (2), where ${\left(\left[y_{1},y_{2},y_{3}\right]\right)}_{\text{concat}}$ is the stacking of *Y_i_* in concatenation. $f_{ReLU}\left(\cdot \right)$ is the ReLU activation function applied after the stacking.

(1) $$Y_{j}=\{\begin{array}{c} y_{1}=f_{c}\left(f_{cb}\left(P_{i}\right)\right)\\ y_{2}=f_{cb}\left(f_{cbl}\left(y_{1}\right)\right)\\ y_{2}=f_{cb}\left(f_{cbl}\left(f_{cbl}\left(y_{1}\right)\right)\right) \end{array}$$

(2) $${\dot{P}}_{i}=f_{c}(f_{ReLU}\left(\left[y_{1},y_{2},y_{3}\right]{)}_{concat}\right)$$

#### 3.2.2 General architecture of WeedNet-R

**Figure 5:**
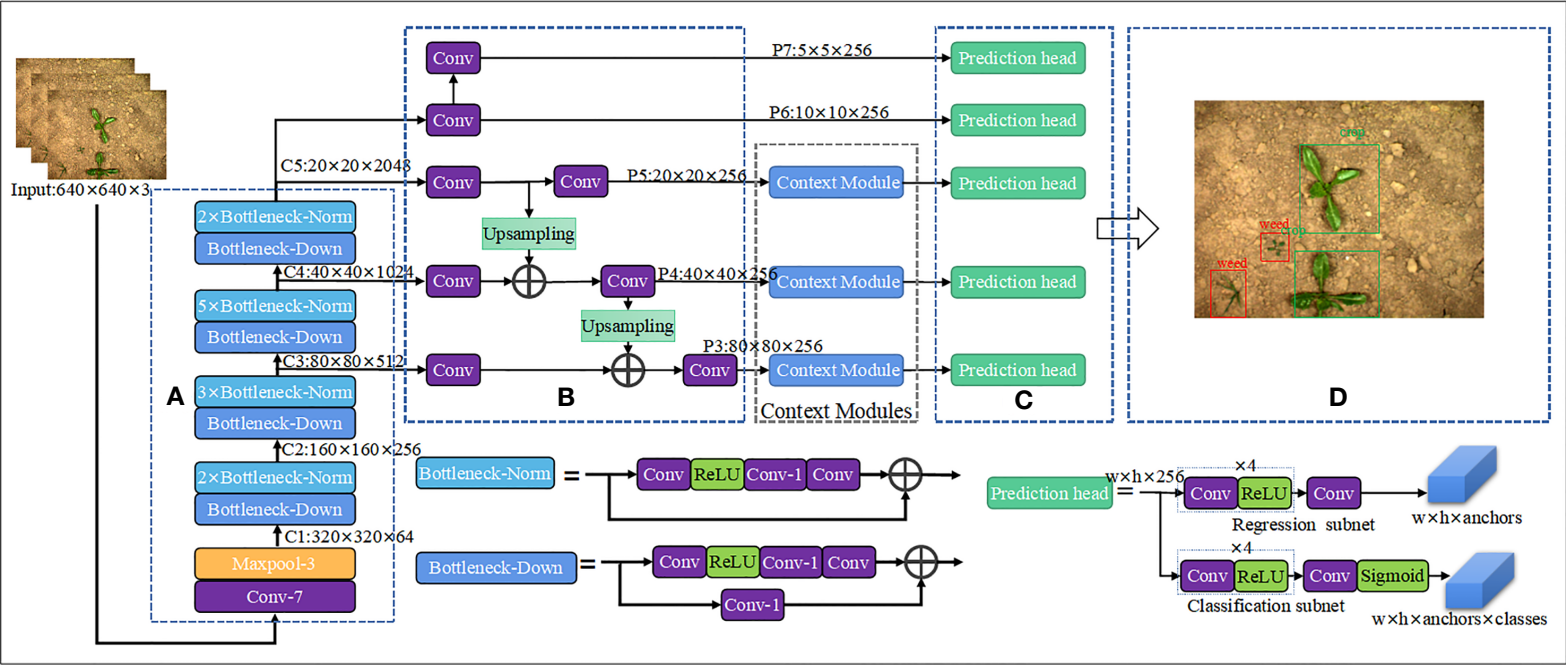
Overall architecture of WeedNet-R: **(A)** Backbone, **(B)** FPN, **(C)** Classification and regression sub-nets, **(D)** Outputs.

Based on RetinaNet and context modules, a one-stage object detection network, the proposed WeedNet-R weed detection network is enhanced in terms of its sophistication and applicability. As seen in **Figure 5**, the WeedNet-R consists of (A) the feature extraction backbone (Backbone), (B) the multi-scale feature pyramid network (FPN), (C) the classification sub-net and regression sub-net, (D) the outputs.

The WeedNet-R uses ResNet50 as the foundation for feature extraction, and the C2-C5 feature layers extracted by ResNet50 are given to the FPN to generate five feature maps P3-P7 with varying scale sizes. Lastly, P3-P7 feature maps are fed into the classification and regression sub-nets for object classification and bounding box regression, respectively. WeedNet-R’s classification and regression sub-nets share the same network weight parameters to reduce the size of the model. Additionally, three context modules are introduced between the three bottom layers (P3-P5) of the FPN and the classification and regression sub-nets in WeedNet-R in order to fuse context information with various receptive field sizes from different levels. In **Figure 5**, *w* and *h* denote the width and height of feature map respectively, *anchors* is number of anchors assigned for each spatial position of feature maps and *classes* is number of object classes.

#### 3.2.3 Loss function

The loss function in WeedNet-R is defined as Equation (3). $N_{pos}$ is the number of positive samples where the prior anchors match the ground-truth labels, *i* is any positive or negative samples, and *j* denotes any positive sample. $L_{total}$ is the total loss function of WeedNet-R. $L_{cls}$ is the classification loss function. $L_{reg}$ is the regression loss function.


(3) $$L_{total}\left(\left\{P_{i}\right\},\left\{t_{i}\right\}\right)=\frac{1}{N_{pos}}\sum_{i}L_{cls}^{i}+\frac{1}{N_{pos}}\sum_{j}L_{reg}^{j}$$

The classification loss function ( $L_{cls}$ ) is calculated according to the focal loss of Equation (4), where $\alpha_{t}$ and $p_{\text{t}}$ are defined as Equation (5) and Equation (6), respectively. The hyperparameter of α∈[0,1] is a weight factor to balance the weights of positive and negative samples, the *p* represents the prediction probability that the sample matches the ground truth. $log\left(p_{\text{t}}\right)$ is the cross-entropy function as Equation (7). A factorization ${\left(1-p_{t}\right)}^{\gamma }$ consisting of another hyperparameter of *γ* and $p_{\text{t}}$ is used to balance the weight of positive and negative samples in the training process. The larger the value of *γ*, the larger the proportion of the loss of the simple samples in the total loss.


(4) $$L_{cls}=-\alpha_{t}{\left(1-p_{t}\right)}^{\gamma }log\left(p_{t}\right)$$

(5) $$\alpha_{t}=\{\begin{array}{c} \alpha \;\;\;\;if\;y=1\\ 1-\alpha \;\;\;otherwise \end{array}$$

(6) $$p_{t}=\{\begin{array}{c} p\;\;\;\;if\;y=1\\ 1-p\;\;\;otherwise \end{array}$$

(7) $$log\left(p_{t}\right)=\{\begin{array}{c} -log\left(p\right)\;if\left(y=1\right)\\ -log\left(1-p\right)\;otherwise \end{array}$$

The regression loss ( $L_{reg}$ ) represents the smooth L1 loss of the bounding box regression, which is shown in Equation (8). Here, $t_{i}=\left[t_{x},t_{y},t_{w},t_{h}\right]$ and $t_{i}^{*}=\left[t_{x}^{*},t_{y}^{*},t_{w}^{*},t_{h}^{*}\right]$ represent the center coordinates, width and height of the predicted bounding box and the ground-truth bounding box, respectively. The definition of $smooth_{L1}$ is shown in Equation (9).


(8) $$L_{reg}=\sum_{i\epsilon \left(x,y,w,h\right)}smooth_{L1}\left(t_{i}-t_{i}^{*}\right)$$

(9) $$smooth_{L1}\left(x\right)=\{\begin{array}{c} 0.5x^{2}\;\;\;\;\;\;if\left|x\right|<1\\ \left|x\right|-0.5\;\;otherwise \end{array}$$

#### 3.2.4 Untuned warmup schedule

**Figure 6:**
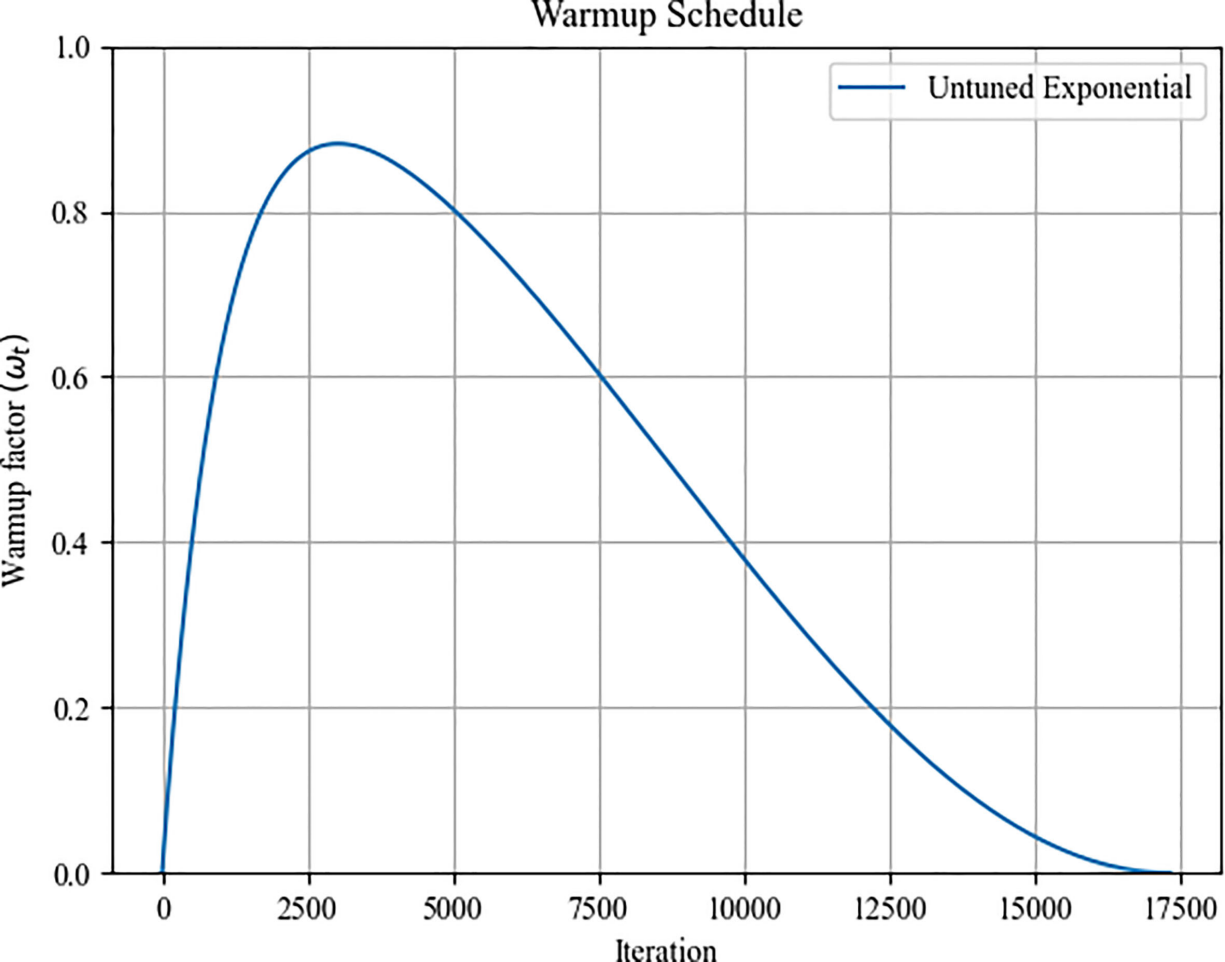
The training schedule learning rate curve.


Here, an untuned exponential warmup schedule is utilized to alter the learning rate during the initial phase of training. **Figure 6** depicts the adjustment of the remaining training period’s learning rate using the cosine annealing process. This adjustment affects the entire training period. Equation (10) determines the number of training steps consumed by the untuned exponential warmup routine throughout the training period. *β_2_* is the Adam optimizer second-momentum coefficient, which takes the default value of 0.999. The learning rate factor *ω(t)* of the untuned exponential warmup schedule is calculated by Equation (11) The final learning rate *lr(t)* of Adam optimizer is calculated by Equation (12), which is the product of *ω(t)* and the initial learning rate.

(10) warmup_period=2/(1−β2)

(11) ω(t)=1-exp(-(1-β2)t)

(12) $$lr\left(t\right)=lr_{init}\times \omega \left(t\right)$$

#### 3.2.5 Crop-first non-maximal suppression

There could be a few exceptions for WeedNet-R during test, which may produce repeated predicted bounding boxes for the same object under a specified confidence threshold. To address this issue, we suggested a crop-first, non-maximal suppression technique for removing anticipated bounding boxes that are repeated. The crop-first non-maximal suppression method is similar to traditional non-maximal suppression method for object detection, but the starting-point of which is to limit the likelihood of crops being destroyed inadvertently, weeds suspected of being crops are frequently given precedence during weeding control. The method firstly separates all prediction results into two groups: sugar beet (crop) bounding boxes and weed bounding boxes. The *IoUs* of each sugar beet bounding box relative to all weed bounding boxes are then determined. Lastly, based on the results of the calculations, any predicted bounding boxes for weed with an *IoU* greater than the given threshold are eliminated.

## 4 Experiment settings

### 4.1 Experimental dataset split

At this study, the experimental dataset is randomly divided into a training-validation set and a test set in a ratio of 8:2. One tenth of the training-validation set is randomly partitioned into a validation set, which is used to observe the convergence of the model during training and to identify the best model after training. The remainder of the training-validation set is utilized for model training as the train set. **Table 1** provides a summary of the employed dataset’s information. The training set has 3,466 images, the validation set contains 387 images, and the test set contains 964 images. Moreover, it can be observed that the ratio of weed objects to sugar beet objects in each subset is near to one-to-one, indicating that the category of the data is balanced.

**Table 1:** Dataset split and statistics of different categories.

	Number of images	Number of weeds	Number of sugar beets
Train subset	3466	6641	6750
Valid subset	387	730	779
Test subset	964	1970	1890
Total	4817	9349	9419

### 4.2 Model training and parameter setting

**Figure 7:**
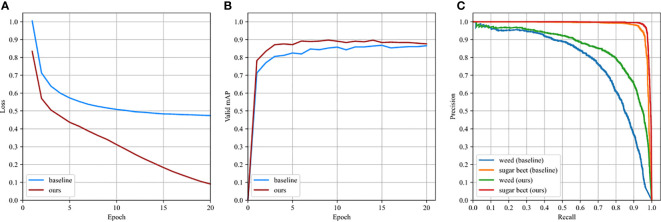
Comparison between baseline and ours: **(A)** loss curve, **(B)** validation *mAP*, **(C)** P-R curve in test subset.

All object detection models in this research were developed using PyTorch 1.2 and Python 3.6 on the Windows 10 operating system. On a PC equipped with a 11 GB Nvidia GeForce GTX2080Ti GPU, a 3.50GHz Intel(R) Core(TM) i9-10920X CPU processor., and 32 GB of main memory, the models were trained and evaluated. To accelerate model convergence, the weights of Resnet50, the backbone network of WeedNet-R, were initialized by an ImageNet-pretrained model. The Adam algorithm was chosen as the model training optimization approach, and the starting learning rate (lr) was set to 0.0001. The initial momentum coefficient article β_1_ was set to 0.9, whereas the second momentum coefficient β_2_ was set to 0.999. The Untuned Exponential Warmup method and the cosine annealing procedure were used to alter the learning rate. The number of samples in each mini-batch was eight, and the model was iterated twenty training epochs. In addition, some training parameters or settings, such as random image flipping, matched RetinaNet (Lin et al., 2020). To compare the performance of WeedNet-R and RetinaNet (baseline), RetinaNet was trained and evaluated using the same settings as WeedNet-R. During model training, the values of the loss function after each epoch iteration were recorded, and model convergence was determined by validating the model on the validation set, as depicted in **Figures 7A, B****)**.

### 4.3 Evaluation metrics

In this article, measures such as mean average precision (*mAP*), size of model parameters, and forward inference time were used to evaluate the effectiveness of the neural network model, and these metrics were computed at an *IoU* threshold of 0.5. The terms of the *IoU* are specified by Formula (13). The mean average precision (*mAP*) is the mean of the average precision (*AP*) of all categories in a multi-category object detection, as defined in Equation (14).


(13) $$IoU=\frac{area\left(B_{p}\cap^{}B_{gt}\right)}{area\left(B_{p}\cup^{}B_{gt}\right)}$$

(14) $$mAP=\frac{1}{n}\sum_{i=1}^{n}AP_{i}$$

where $area\left(B_{p}\cap^{}B_{gt}\right)$ is the area of intersection of the predicted bounding box (*B_p_*) and the groundtruth bounding box (*B_gt_*). $area\left(B_{p}\cup^{}B_{gt}\right)\;$ is the area of the union of *B_p_* and *B_gt_*. Average Precision (*AP*) is related to *precision* and *recall*, which are calculated by Equation (15) and Equation (16), respectively. Where *T_p_* (true positive) represents the number of predicted results with *IoU* >threshold, *F_P_* (false positive) represents the number with *IoU* ≤ threshold. *F_n_* (false negative) represents the number of true bounding boxes not detected. The confusion matrix for *T_p_*, *F_n_*, and *F_p_* is shown in **Table 2**.

**Table 2:** Definition relationships between predicted and true values.

Ground Truth	Predicted	
	Positive	Negative
Positive	True Positive (*T_p_*)	False Negative (*F_n_*)
Negative	False Positive (*F_p_*)	True Positive (*T_n_*)


(15) $$Precision=\frac{T_{p}}{T_{p}+F_{p}}$$

(16) $$Recall=\frac{T_{p}}{T_{p}+F_{n}}$$

According to the prediction confidence, a set of recall and accuracy regarding the results of the forecast are calculated individually for various confidence thresholds. Obtaining the P-R curve p(r) using recall as the horizontal axis and precision as the vertical axis. The final step in calculating the average accuracy of a single category is to solve the integral between the P-R curve and the horizontal axis, as shown in Equation (17).


(17) $$AP=\int_{0}^{1}p\left(r\right)dr\;$$

### 4.4 Comparison with the state-of-the-art object detection models

To further demonstrate the efficacy and superiority of the enhanced model, the performance of WeedNet-R was compared to that of other advanced object detection methods, with the exception of the baseline model (RetinaNet), under identical experimental conditions. Faster R-CNN (Ren et al., 2017), SSD (Liu et al., 2016), YOLOv3 (Redmon and Farhadi, 2018), YOLOv4 (Bochkovskiy et al., 2020), CenterNet (Zhou et al., 2019), YOLOX (Ge et al., 2021). YOLOv7 (Wang et al., 2022) are the models compared. The *AP*, *mAP*, number of model parameters, forward inference time, etc. measured on a test set from sugarbeet2016 serve as the primary comparative performance indicators. In consideration of the modest variation in image input size between detectors, the input image size for training and testing is equally scaled to be 640 by 640 pixels or near to that size. In addition, both Faster R-CNN and CenterNet utilise ResNet50 and VGG16 as their extraction backbone. Yolov3 and YOLOv4 both utilise Darknet53 with 53 layers as their backbone. And the standard CSPDarknet53 was used as backbone of YOLOX. The aforesaid settings make these comparison models match an equivalent level of model parameters as the WeedNet-R, thus ensuring the fairness of the comparison.

## 5 Experiment results

### 5.1 Detection performance on dataset

**Figure 7C** depicts the P-R curves of the models on test data. The validation and test set images were utilised to evaluate the performance of the respective models. **Table 3** displays the evaluation’s results WeedNet-R only increased the number of parameters by 4.4% compared to the baseline model, while the *mAP* metric in the validation set improved by 2.93%, and the average accuracy (*AP*) for weed and sugar beet detection rose by 4.61% and 1.21%, respectively. In the meantime, the test set reflected the increased detection performance of the proposed model: the *mAP* improved by 4.65% to 92.30%, while the average accuracy of weed and sugar beet identification improved by 8.01% to 85.70% and 1.29% to 98.89%, respectively. Due to the fact that the P-R curve reflects the variable relationship between recall and accuracy at different confidence thresholds, the better the performance of the detector is represented, the closer the shape contained by the P-R curve and the coordinate axis is to a square. **Figure 7C****)** shows that the precision of WeedNet-R is marginally higher than that of the baseline model for sugar beet plant detection and significantly higher than that of the baseline model for weed detection at the same recall rate. The aforementioned findings demonstrate that our improved weed detection system outperforms RetinaNet.

**Table 3:** Comparison of the detection performance of the RetinaNet model (baseline) before improvement and WeedNet-R.

method	valid set (%)			test set (%)			parameters	inference times (ms)
	weed *AP*	sugar-beet *AP*	*mAP*	weed *AP*	sugar-beet *AP*	*mAP*		
RetinaNet	76.38	97.27	86.82	77.69	97.61	87.65	36.4M	66.4
WeedNet-R	80.99	98.51	89.75	85.70	98.89	92.30	38.0M	52.8

WeedNet-R outperforms the other six detection algorithms on the test set in terms of both the *AP* in individual categories and the *mAP* when compared to other sophisticated object detectors, as shown **Table 4**. The detection capabilities of the suggested method are much superior to those of previous methods. WeedNet-R’s *mAP* is 10.65% greater than the most inaccurate Fast R-CNN. Compared with the latest SOTA object detector YOLOv7, our approach’s *mAP* is 0.8% higher than it. It is worth noting that YOLOv7 applied some complex data augmentation approaches such as mosaic during training, but weedNet-R did not apply complex data augmentation approach. In addition, WeedNet-R has a lesser number of parameters than the YOLO series of algorithms (YOLO v3, YOLO v4, and YOLOX) and a somewhat greater number of parameters than SSD CenterNet and YOLOv7, which are noted for their simple architecture. Consequently, our strategy achieves optimal detection accuracy while also ensuring more acceptable model parameters. It is worth noting that the inference speed of all the models utilized in our experiments was slow, primarily due to the utilization of a relatively older graphics card from the 1080 series. This older graphics card exhibits a significant performance gap compared to the latest advanced graphics cards available in the market.

**Table 4:** Comparison of the detection performance of WeedNet-R with different target detectors.

	backbone	weed *AP*	sugar-beet *AP*	*mAP*	parameters	inference time (ms)
Faster R-CNN	Resnet50	66.28%	97.02%	81.65%	28.3M	80.1
SSD	VGG16	69.16%	97.11%	83.14%	23.7M	27.2
YOLOv3	Darknet53	82.82%	96.99%	89.90%	61.5M	62.7
YOLOv4	CSPDarknet53	81.60%	97.36%	89.48%	63.9M	40.3
CenterNet	Resnet50	80.79%	97.06%	88.93%	32.7M	42.2
YOLOX	CSPDarknet53	81.90%	90.88%	86.39%	54.2M	32.1
YOLOv7	E-ELAN	84.20%	98.80%	91.50%	36.5M	21.1
WeedNet-R	Resnet50	85.70%	98.89%	92.30%	38.0M	52.8

### 5.2 Ablation experiment results

As indicated in **Table 5**, a number of ablation experiments were conducted to determine the efficacy of each modified module. According to the findings of the ablation experiments, the addition of context module×5 and context module×3 to RetinaNet enhances its *mAP* by 2.17 and 1.99 percentage points, respectively. Combinations of context module×5 and untuned warmup that are added to RetinaNet increase its *mAP* metrics by 4.41%. Adding context module×3 and untuned exponential warmup learning rate adjustment approach to RetinaNet results in WeedNet-R, which achieves the maximum *mAP* value of 92.30%. Specifically, its *AP* scores for weed detection are improved by 8.01% to 85.70%. In conclusion, adding three context modules or five context modules is effective, although the trick with three context modules yields slightly higher detection accuracy with fewer parameters.

**Table 5:** Ablation experiments results.

	performance(%)				
	RetinaNet				WeedNet-R (ours)
+context module ×5	–	√	–	√	–
+context module ×3	–	–	√	–	√
+untuned warmup	–	–	–	√	√
weed *AP*	77.69	81.40	81.14	85.19	85.70
sugar beet *AP*	97.61	98.24	98.15	98.94	98.89
*mAP*	87.65	89.82	89.64	92.06	92.30

context module×5: Add a context module after each of the P3~P7 layers of the FPN network output. context module×3: Add a context module after each of the P3~P5 layers of the FPN network output. untuned warmup: Set untuned exponential warmup schedule during model training. “-” indicates that the method does not contain the corresponding module, and “√” indicates that the method contains the corresponding module.

### 5.3 Visualization

**Figure 8:**
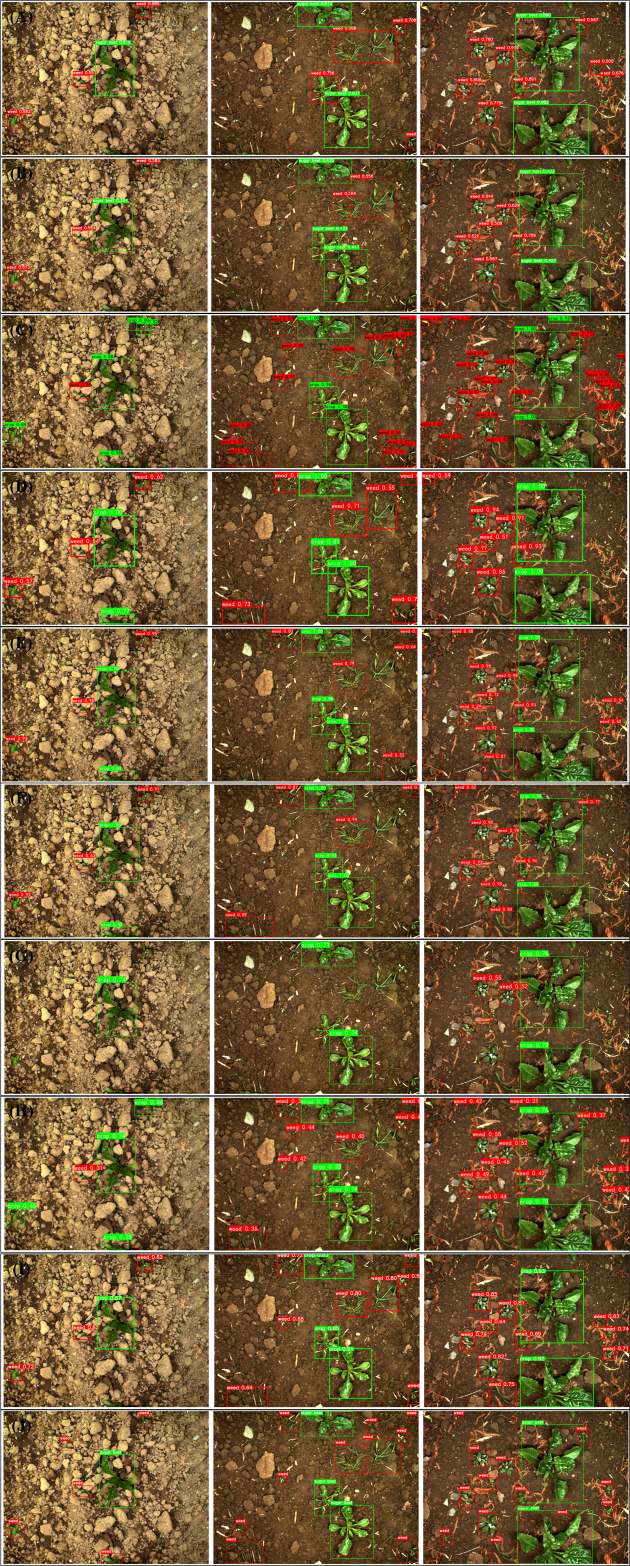
Detection results of different models: **(A)** WeedNet-R, **(B)** RetinaNet, **(C)** Faster R-CNN, **(D)** SSD, **(E)** YOLOv3, **(F)** YOLOv4 **(G)** CenterNet, **(H)**YOLOX, **(I)** YOLOv7, **(J)** Ground-truth.

This section validates the usefulness of WeedNet-R for real input images by conducting visualization experiments and an analysis of the test dataset. As depicted in **Figures 8A–I****)** are the prediction results of the experimental models for three representative images from the test dataset with varying background complexity, and **Figure 8J** is the ground truth. The complexity of the backdrop and the number of objects in the three selected images increase from left to right in order to evaluate the performance of various algorithms under varying scenarios. As shown in **Figures 8A, B****)**, compared to RetinaNet, the proposed method provides more accurate prediction results, greater confidence in the classification of the items within the predicted bounding boxes, and more precise placements for the predicted bounding boxes. Compared to other sophisticated detection algorithms, sugar beet identification performance was comparable, with the exception of CenterNet, which made more incorrect predictions. For weed detection, Faster R-CNN suffers from a severe case of repeated prediction, and SSD is unsuitable for small objects. Both YOLOV3 and YOLOV4 have instances of missing marijuana detection. The CenterNet makes inaccurate predictions of weeds and has poor trust in the accuracy of its predictions. The recently popular YOLOX and YOLOv7 algorithms have a decent detection performance, yet there have been instances were weeds were not detected.

### 5.4 Optimization for repeated prediction boxes

**Figure 9:**
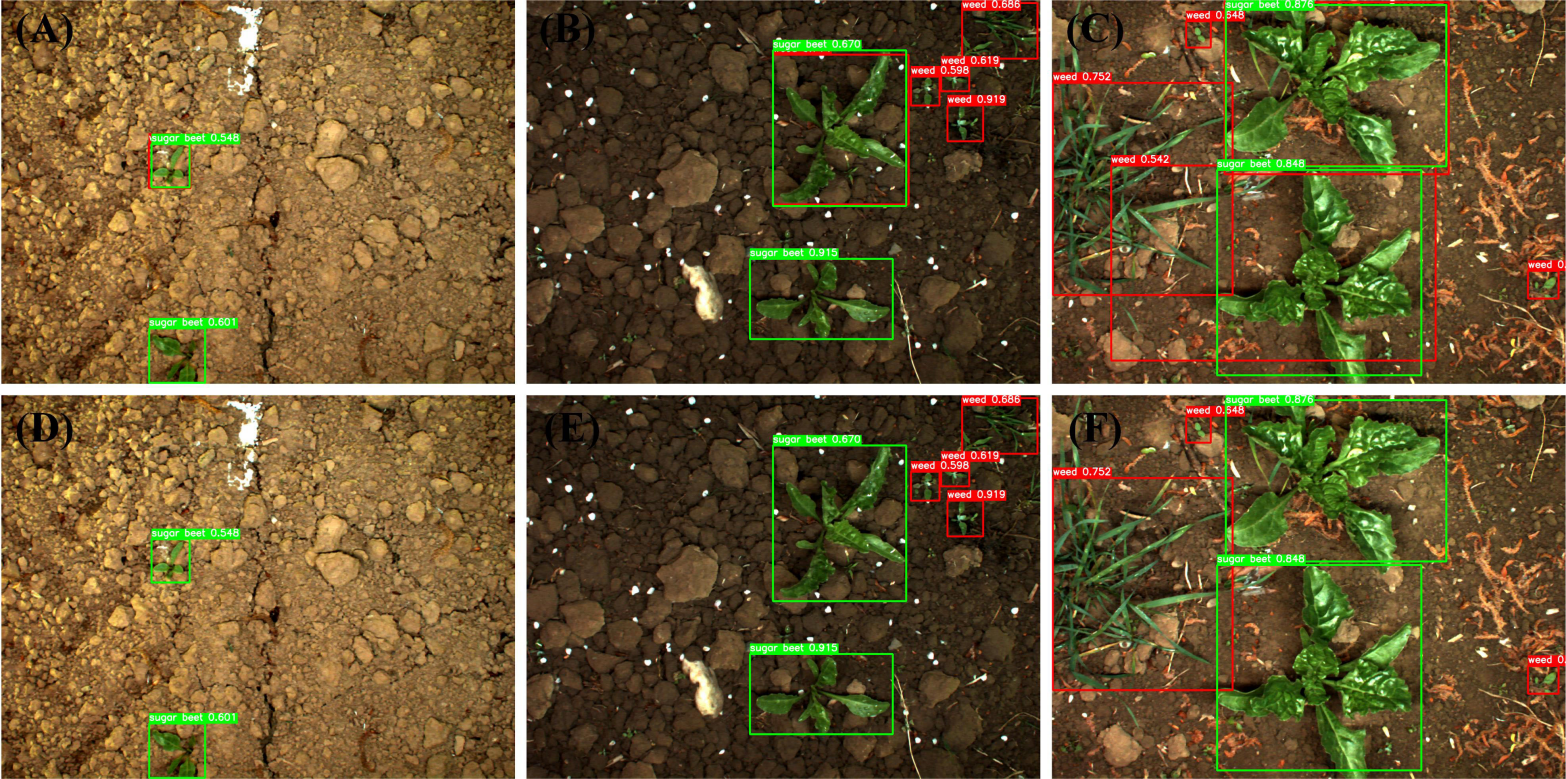
Comparison of prediction results before and after crop-first non-maximal suppression method: **(A-C)** are before, **(D-F)** are after.

The prediction results before and after crop-first non-maximal suppression method are shown in Figures **Figures 9A–F**. (**Figure 9A****)** illustrates a limited number of exceptions in prediction results, which produces repeated predicted bounding boxes for the same object under a confidence level of 0.5. This problem is mitigated by applying crop-first non-maximal suppression method as shown in **Figure 9B****)**. Here, the *IoU* threshold for crop-first non-maximal suppression is set to 0.5. As demonstrated in **Table 6**, this strategy enhances weed detection precision by 0.2%, but has no influence on the detection accuracy and recall of sugar beet. Consequently, the crop-first non-maximal suppression method accomplishes the goal of eliminating duplicate anticipated bounding boxes and minimizing the possibility of false crop removal.

**Table 6:** Comparison before and after using the crop-first non-maximal suppression method.

	weed		sugar beet	
	precision	recall	precision	recall
before	76.5%	82.5%	97.0%	96.5%
after	76.7%	81.9%	97.0%	96.5%

### 5.5 Validation on other public weed dataset

To validate the effectiveness of our improved method, we conducted additional experiments using a another weed dataset (Ravirajsinh, 2020) containing while keeping the experimental configurations consistent. The results are presented in **Table 7**, showcasing the performance of our proposed approach on publicly available weed datasets. Notably, our WeedNet-R model achieved an *mAP* metric of up to 85.26%. It is important to note that the improvements in detection performance of WeedNet-R, compared to the original RetinaNet, were relatively modest on this new weed dataset, with a increase of 0.57% in the *mAP* metric.

**Table 7:** Comparison between the RetinaNet and WeedNet-R on anther public weed dataset.

method	weed *AP*(%)	sesame *AP*(%)	*mAP*(%)
RetinaNet	86.21	83.16	84.69
WeedNet-R	86.91 + ^0.70^	83.61 + ^0.45^	85.26 + ^0.57^

### 5.6 Discussion

Experiments comparing our proposed approach to other sophisticated object detection algorithms demonstrate that the suggested algorithm has the highest average precision of individual categories and the highest total *mAP* metrics, as well as the highest detection accuracy. Despite the fact that WeedNet-model R’s parameters are greater than those of the original RetinaNet, the average detection time is shorter. This is due to the fact that the less precise RetinaNet generates more inaccurate predictions during detection, which increases the time required for post-processing actions such as non-maximal suppression. Ablation studies confirmed the efficacy of our enhancements to RetinaNet. Experiments indicate that the optimal detection performance is achieved by adding a context module after each of P3 to P5 of the FPN outputs and configuring an untuned exponential warmup schedule during model training. Adding a context module to each of the P3 to P5 levels of the FPN reduces several parameters of the model and somewhat increases model recognition accuracy compared to adding a context module to each layer of the FPN outputs. This may be because the P6-P7 layers of the FPN are part of the high-level feature maps, which include sufficient deep context information to identify huge objects.

In addition, under natural light, the colour, morphology, and texture of sugar beet plants (especially early sugar beet seedlings) closely resemble those of field weeds. This resemblance exacerbates the difficulty of differentiating weeds from crops and is the primary cause of the model’s misclassification of identified objects. In reality, this frequently shows as misclassification of objects within the projected bounding boxes or the generation of several predictions for the same object. We presented a crop-first non-maximum suppression strategy for a problem involving repeated predicted bounding boxes for the same object. Objects projected to be both weed and crop repeatedly at the same location are classed as crop in order to reduce the chance of crops being removed in error.

The model we proposed demonstrates effective performance on other publicly available weed datasets, albeit with a relatively modest increase of 0.57% in the mean average precision (*mAP*) metric compared to the baseline. We attribute this outcome, at least in part, to the limited number of images available in evaluated dataset. Building a robust model with a small number of images poses significant challenges. The scarcity of large-scale publicly available weed datasets remains a common obstacle in the domain of weed detection utilizing deep learning approaches. To overcome this challenge, future endeavors should focus on the acquisition and curation of larger and more diverse weed datasets. Therefore, we have made our annotated weed dataset based on Sugarbeet2016 publicly available to support the research community and facilitate future advancements in this field. Our annotated dataset comprises 4,817 images and 18,768 annotations, making it one of the most extensive bounding box-based datasets for weed detection.

## 6 Conclusion

Detecting and identifying weeds in the field is a crucial step in attaining autonomous weed management. While the remarkable resemblance in color, morphology, texture, and other features between weeds and crops in the field under natural lighting conditions increases the complexity of machine vision-based weed detection. Theoretical and methodological developments in deep learning have produced new tools for visual identification problems, such as weed detection. Due to the complexity of weed detection tasks in the field, deep learning-based approaches for weed detection continue to be of significant scientific relevance. In this research, we present an enhanced detection model, WeedNet-R, which is based on RetinaNet and has greater detection accuracy than the original model and other sophisticated object detectors. WeedNet-R has the highest *mAP* for weed detection in sugar beet fields at 92.30%.

In this study, we relate the lack of detection accuracy of the baseline model to the insufficient size of its effective receptive field. In order to increase the effective receptive field of the feature extraction layers, context modules are added to the neck structure of RetinaNet. During model training, an untuned exponential warmup schedule is implemented in order to improve the optimal solution search capability. The *mAP* of WeedNet-R proposed in this article was enhanced by 4.65% as compared to the original RetinaNet as a result of the aforementioned enhancements. With only a little improvement in model parameters, the accuracy of weed detection increased by 8.01% to 85.70%, and the accuracy of sugar beet plant recognition increased by 1.2% to 98.89%. In addition, the crop-first non-maximal suppression strategy we presented reduces the few occurrences in which the same object is predicted many times by the model. The detection performance of the proposed approach is superior to that of other algorithms in the SugarBeet2016 dataset, but there is still a little room for improvement in weed detection. Therefore, continuing to optimize the structure of our model is our future efforts. And because larger image dateset would be beneficial for training of convolutional neural networks, the model’s performances may be further optimized by obtaining more weed images. In addition, the size of model’s parameters is key to the performance of model forward inference. Perhaps it is well worth considering to boost the model’s detection speed by refining the model’s backbone or implementing an anchor-free strategy for boosting the model’s detectionsssss speed. In conclusion, pursuing a more precise and faster weed identification model to deal with the complex farming environment will be the primary focus of our future work.

## Data availability statement

The datasets presented in this study can be found in online repositories. The names of the repository/repositories and accession number(s) can be found in the article/**Supplementary Material**.

## Author contributions

ZG: Software, Data curation, Resources, Visualization, Writing – original draft, Writing – review and editing. YL: Conceptualization, Methodology, Writing – original draft, Writing – review and editing. HG: Validation, Super-vision, Writing – review and editing. XL: Validation Formal analysis, Investigation. MZ: Validation, Investigation, Visualization. All authors contributed to the article and approved the submitted version.

## Footnotes

1 Available at https://github.com/GOOJJJ/WeedNet-R/
