# Supplementary material for: WeedNet-R: a sugar beet field weed detection algorithm based on enhanced RetinaNet and context semantic fusion
Source: Front Plant Sci. 2023 Jul 24;14:1226329. doi: 10.3389/fpls.2023.1226329 (PMC10408303; doi:10.3389/fpls.2023.1226329)
Supplement: Supplementary file 1 [file DataSheet_1.pdf]

Supplementary Material

To assess the performance gap between our proposed WeedNet-R and state-of-the-art detectors, we trained the latest SOTA model YOLOv7[1] on Sugar-beet2016 dataset with the same hyper-parameters as the official implementation. However, our proposed model only employed simple data augmentation techniques such as image flipping, whereas YOLOv7 utilized complex techniques such as mosaic. Therefore, we fit and evaluated two versions of YOLOv7, one with complex data augmentation and one without. **Supplementary Figure 1** illustrates the evaluation results on the validation set during training for both cases.

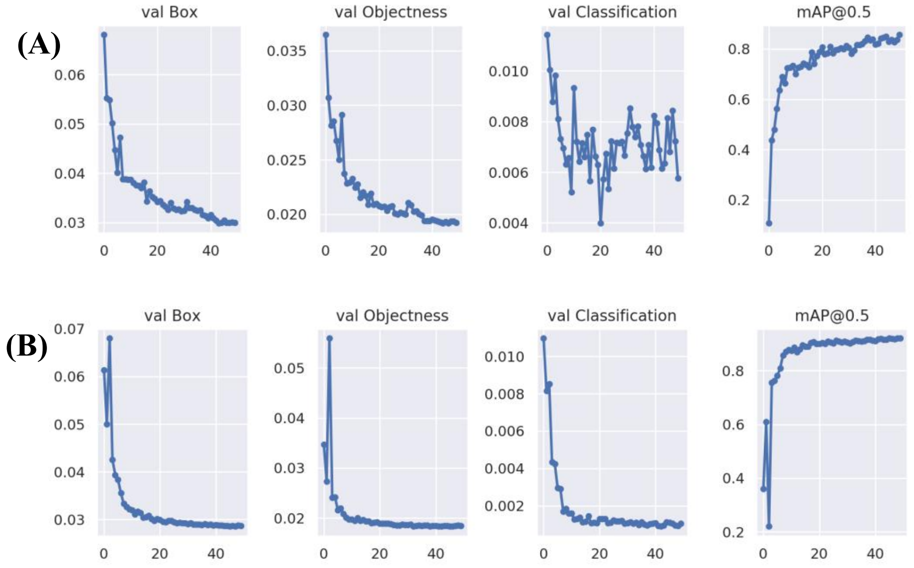

Supplementary Figure 1. The validation results for YOLOv7 models training:(A) with simple data augmentation, (B) with complex data augmentation.

Supplementary Table 1. Comparison of the detection performance of WeedNet-R with SOTA detector YOLOv7.

	backbone	data augmentation	weed AP	sugar-beet AP	mAP	parameters	inference time (ms)
YOLOv7	E-ELAN	simple	73.70%	92.5%	83.10%	36.5M	20.8
YOLOv7	E-ELAN	complex	84.20%	98.80%	91.50%	36.5M	21.1
WeedNet-R	Resnet50	simple	**85.70%**	**98.89%**	**92.30%**	38.0M	52.8

As shown in **Supplementary Table 1**, our proposed approach outperformed YOLOv7 in terms of AP and mAP metrics. Specifically, WeedNet-R's mAP was 0.8% higher than that of YOLOv7 when

[1] Wang, C. , Bochkovskiy, A. , and Liao, H. . (2022). Yolov7: trainable bag-of-freebies sets new state-of-the-art for real-ti me object detectors. arXiv e-prints.

using complex data augmentation. Additionally, our approach's mAP was 9.2% higher than YOLOv7 without complex data augmentation during training. Although YOLOv7 is a faster object detector, the comparison experiments highlighted the advantages of our proposed approach in terms of detection accuracy.

To validate the effectiveness of our improved method, we repeated the previous experiments on another weed dataset[1] with the same experimental configurations. As presented in **Supplementary Table 2** and **Supplementary Figure 2**, our proposed approach performs well on other publicly available weed datasets, with WeedNet-R achieving an mAP metric of up to 85.26%. However, the improvements in detection performance of WeedNet-R compared to the original RetinaNet are limited on this new weed dataset, with an increase of only 0.57% in the mAP metric. We attribute this outcome, in part, to the small number of images in the new dataset. Developing a robust model with a limited number of images is challenging. In fact, the lack of large public weed datasets is a common obstacle in weed detection using deep learning. To address this challenge, future efforts should focus on the acquisition and curation of larger and more diverse weed datasets. Despite the challenges, the potential benefits of weed detection using deep learning are vast, and continued efforts in this area could have significant implications for agricultural sustainability and weed management.

Supplementary Table 2. Comparison of the detection performance of the RetinaNet and WeedNet-R on anther public weed dataset[2].

method	weed AP(%)	sesame AP(%)	mAP(%)
RetinaNet	86.21	83.16	84.69
WeedNet-R	**86.91**$^{+0.70}$	**83.61**$^{+0.45}$	**85.26**$^{+0.57}$

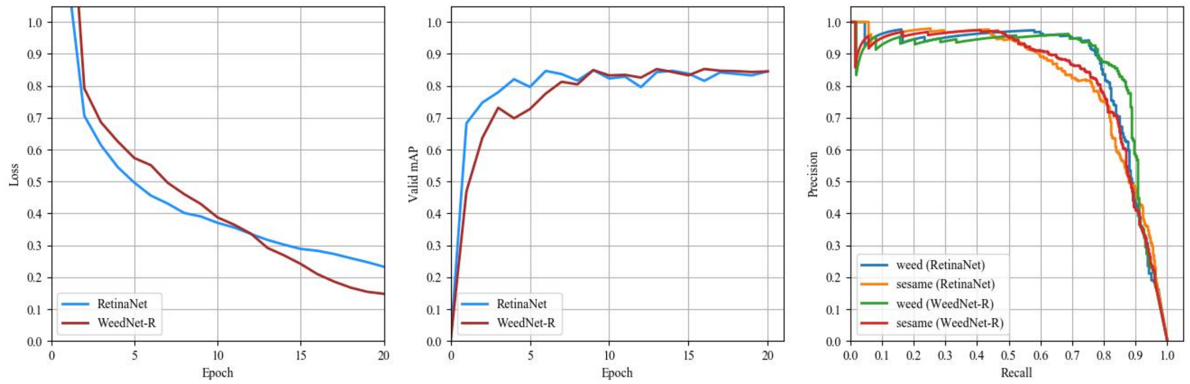

(A) **(B)** **(C)**

Supplementary Figure 2. Comparison of RetinaNet and WeedNet: (A) loss curve, (B) evaluate mAP, (C) P-R curve in test set.

[2] Ravirajsinh, D,. (2020) crop and weed detection data with bounding boxes. https://www.kaggle.com/datasets/ravirajsinh45/crop-and-weed-detection-data-with-bounding-boxes.

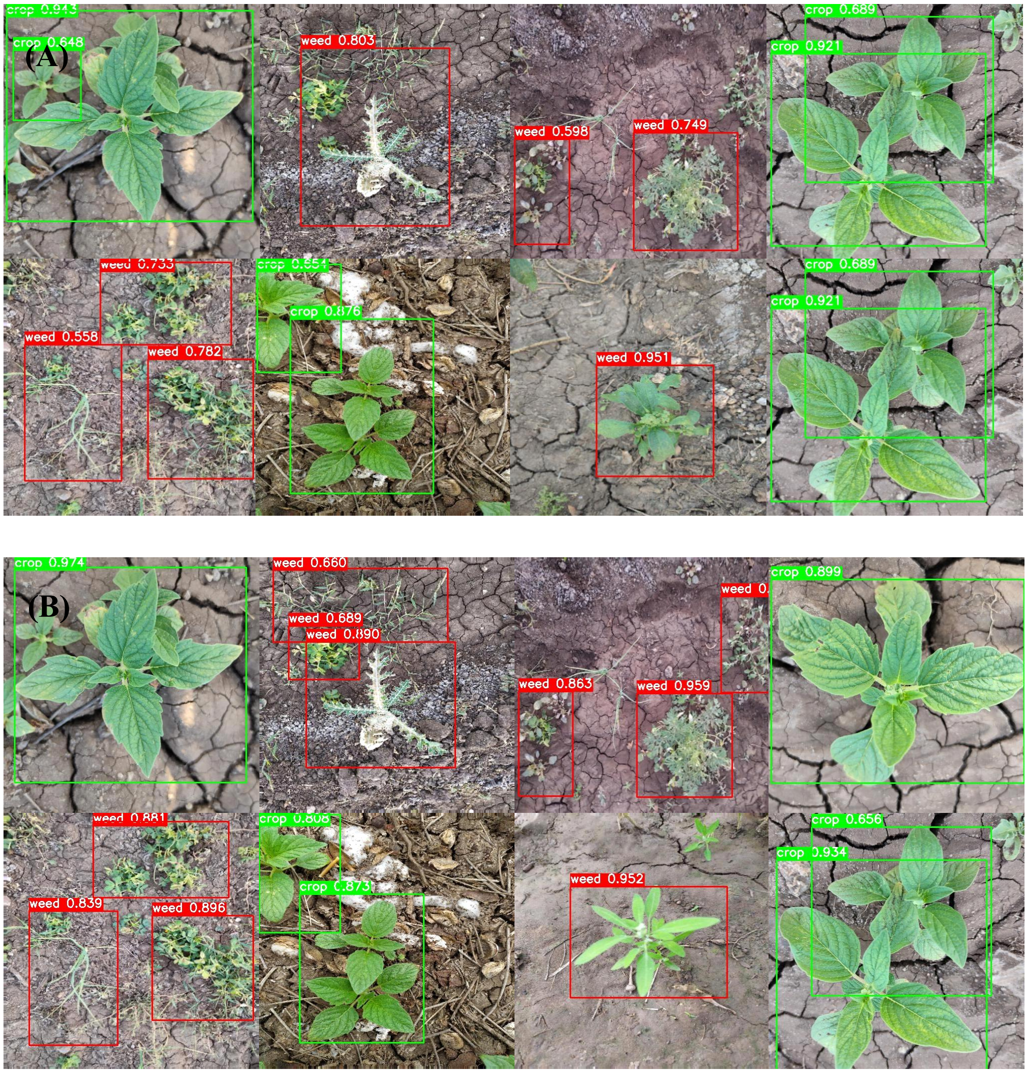

Supplementary Figure 3. Detection results with a score threshold of 0.5: (A) RetinaNet, (B) WeedNet-R
